# Molecular profiling of lipid droplets inside HuH7 cells with Raman micro-spectroscopy

**DOI:** 10.1038/s42003-020-1100-4

**Published:** 2020-07-10

**Authors:** Ashok Zachariah Samuel, Rimi Miyaoka, Masahiro Ando, Anne Gaebler, Christoph Thiele, Haruko Takeyama

**Affiliations:** 1 Research Organization for Nano & Life Innovation, Waseda University, 513, Wasedatsurumaki-cho, Shinjuku-ku Tokyo, 162-0041 Japan; 2 Department of Life Science and Medical Bioscience, Waseda University, 2-2 Wakamatsu-cho, Shinjuku-ku Tokyo, 162-8480 Japan; 3 JST, PRESTO, 4-1-8 Honcho, Kawaguchi Saitama, 332-0012 Japan; 4 LIMES Life and Medical Sciences Institute, University of Bonn, Carl-Troll-Strasse 31, 53115 Bonn, Germany; 5 Computational Bio Big-Data Open Innovation Laboratory, National Institute of Advanced Industrial Science and Technology and Waseda University, 3-4-1 Okubo, Shinjuku-ku Tokyo, 169-8555 Japan; 6 Insituture for Advances Research of Biosystem Dynamics, Waseda Research Institute for Science and Engineering, Tokyo, Japan

**Keywords:** Raman spectroscopy, Molecular imaging

## Abstract

Raman imaging has become an attractive technology in molecular biology because of its ability to detect multiple molecular components simultaneously without labeling. Two major limitations in accurately accounting for spectral features, *viz*., background removal and spectral unmixing, have been overcome by employing a modified and effective routine in multivariate curve resolution (MCR). With our improved strategy, we have spectrally isolated seven structurally specific biomolecules without any post-acquisition spectral treatments. Consequently, the isolated intensity profiles reflected concentrations of corresponding biomolecules with high statistical accuracy. Our study reveals the changes in the molecular composition of lipid droplets (LDs) inside HuH7 cells and its relation to the physiological state of the cell. Further, we show that the accurate separation of spectral components permits analysis of structural modification of molecules after cellular uptake. A detailed discussion is presented to highlight the potential of Raman spectroscopy with MCR in semi-quantitative molecular profiling of living cells.

## Abstract (web summary)

Samuel, Miyaoka et al. investigate the changes in the molecular composition of lipid droplets inside HuH7 cells and its relation to the physiological state of the cell, using Raman spectroscopy and multivariate curve resolution. This study underscores the importance of separation of spectral components in semi-quantitative molecular profiling of living cells.

## Introduction

Living cells are complex entities with organelles formed by collectively existing molecules performing specific functions. One such seemingly simple but dynamic cell organelle is lipid droplet. Lipid droplets, which are made up of lipid-rich core surrounded by monolayer of phospholipids, are generally thought to be storage organelles^1,2^. There is a great amount of interest in molecular profiling of LDs inside cells as more and more evidences reveal its functional role^3^ beyond storage of molecules^4,5^. LDs play substantial, but yet not completely understood, role in diseases; for instance, fatty liver disease, diabetes^6^, hepatitis C virus (HCV) infection^7^, and cancer^8^. LDs have been shown to play substantial roles in viral assembly during virus replication^7^, as storages sites for specific histones^9^, mediators in transcription^10^, phosphatidylcholine synthesis^11^ etc. These studies indicate a larger role LDs play in sustaining life. Molecular profiling of LDs is hence an extremely important aspect of molecular biology^12^.

Fluorescence microscopy is extensively used in molecular biology for investigating distributions of molecules and organelles, including LDs, inside single cells. Lipid-selective fluorescent molecules are generally used to light-up LDs inside cells (Nile Red^13^ and BODIPY^14^). However, a study of the distribution of several different molecules inside LDs would require each of them to be selectively labeled. In such efforts, it is necessary to avoid lager molecular structural alterations of the probe-molecule such that the biological functional equivalence of the labeled species is largely unaffected compared to its natural analogue. ‘Alkyne tagging’^15,16^ has been demonstrated as one of the smallest possible ‘tagging’ methods for subsequent fluorescent labeling. Supporting this argument, esterification of alkyne cholesterol in glioblastoma cells were found to proceed efficiently, equivalent to ‘untagged’ cholesterol^15^. Derivatives of fatty acids with strategically extended conjugation also serve as fluorescent molecules with minimal structural alteration for single-cell imaging^17^. However, a critical evaluation of biological behavior of labeled and native molecules inside a cell is very important in the light of extensive use molecular labeling methodology in molecular biology and medicine. This would require selective detection of these derivatives when they are simultaneously present inside the same cell. Earlier such attempts were often unsuccessful^15^. We have fed cells with alkyne-tagged cholesterol both to evaluate accuracy of the analytical method and to test the biological equivalence of natural and tagged cholesterol derivatives.

Raman micro-spectroscopy is well suited for molecular profiling since it can detect multiple molecules simultaneously. Molecules such as proteins, lipids, cholesterol, nucleic acid etc. show distinguishable vibrational spectrum permitting molecular imaging of living cells^18^. However, linear Raman imaging technique has often been criticized for the inferior signal strength compared to the nonlinear analogues such as coherent anti-stokes Raman spectroscopy (CARS) and stimulated Raman scattering (SRS)^19^. But it should be noted that, in order to get full spectral information, CARS and SRS employ high power pulsed lasers and are shown to be detrimental to the biological constituents^20–22^. Use of a narrowband laser pulse instead would allow rapid image acquisition but at the cost of multiplex ability^21^. Further, in CARS, the nonlinear polarization in an inhomogeneous system (cells) along with non-resonant background results in spectrum considerably different from the corresponding Raman spectrum^21^. Local environments, number, shape, and size of scatterers also affect CARS spectrum and intensity leading to difficulty in directly estimating the composition of different molecular species in biological cells^22,23^. Therefore, nonlinear techniques have limitations when it comes to molecular concentration profiling of cells. However, remarkable rapid imaging capabilities of these techniques provide useful information on distribution of lipids in biological cells and tissues^19,24–26^.

Ease of multiplexing is an advantage of Raman spectroscopy over fluorescence and nonlinear Raman techniques. At the same time, due to multiplexing, a composite Raman spectrum of several molecular components is generated from each focal spot during imaging. Therefore, multiplex Raman imaging require efficient data analysis techniques that effects separation of spectral components and their concentration profiles from the composite spectrum. Multivariate curve resolution by alternating least squares (MCR-ALS) combined with singular value decomposition (SVD)^27–30^ is an appropriate multivariate analytical technique that separates interpretable spectral components and their concentration profiles. The efficacy of the method has been demonstrated in earlier studies including several of our own^27–30^. However, when applied to biological systems, this technique encounters a serious problem from highly fluctuating background. This often get manifested as signal-signal, signal-background mixing, and limits spectral unmixing. Therefore, undesirable arbitrary baseline correction and other post-acquisition spectral modifications are routinely performed. In this study we demonstrate a modified MCR routine that allows better signal separation from broad backgrounds with highly improved spectral unmixing. Consequently, we could obtain intensity profiles reflecting statistically accurate compositions of biomolecules that reflected the biological features of the cells and organelles.

In the present investigation, we have applied spontaneous Raman spectroscopy and the improved MCR-ALS routine for molecular profiling of individual lipid droplets inside HuH7 human liver cells. LDs in each cell show small but considerable variations in the concentrations of molecular constituents but a drastic change was observed upon changing the cell-culturing condition. Through detailed analysis, we provide explanation for these changes based on statistically significant variations in the molecular composition of the cell. A detailed discussion of the data analysis to emphasize the potential of Raman-MCR technique in analyzing biological systems is provided in the manuscript.

## Results

Oleic acid is known to affect formation of LDs in hepatic cells^31^. Similarly, structural modifications of LDs in response to cholesterol have fundamental significance in biology of hepatic cells^32^. The way these molecules affect LDs formation and their composition remains unclear. Therefore, understanding molecular changes in LDs under the influence of these molecules is valuable. HuH7 human liver cells were cultured in laboratory using DMEM medium in a chamber with glass bottom (see “Methods” for details). Two different feed conditions (Table 1) were used in the present study: (1) OL-feed condition—culture medium containing oleic acid and alkyne cholesterol (2) CH-feed condition—culture medium containing cholesterol and alkyne cholesterol. Six different HuH7 cells were analyzed in the present study: three cells from each feed condition. Raman images were recorded from all six cells (see “Methods” for details).

**Table 1:** Different feed conditions used for the study.

Name	Second day		Third Day			Cells
	Cholesterol^a^ (µM)	Oleic acid (µM)	Cholesterol (µM)	Oleic acid (µM)	Alkyne Cholesterol^a^ (µM)	
OL-feed		50		50	50	1–3
CH-feed	50		50		50	4–6

^a^The cholesterol and alkyne cholesterol in the culture medium is in the free form and they get enzymatically esterified after cellular uptake (see the discussion).

### Identifying LDs in cells from Raman images

**Fig. 1:**
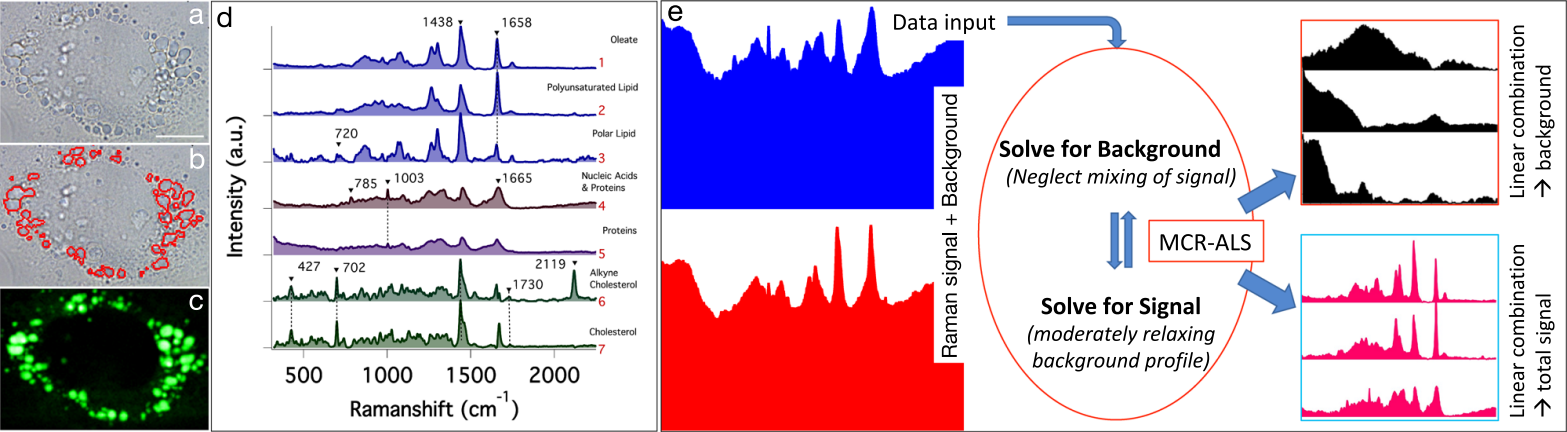
Image analysis and MCR-ALS analysis. **a** White light Image of the cell. Lipid droplets can be identified as globules. Scale bar 10 μm. **b** LD boundaries. **c** Spatial distribution of intensity of alkyne bond stretching mode (2119 cm^−1^) of alkyne cholesterol. **d** Statistically significant spectral components separated in the MCR-ALS analysis. We have assigned them as, 1—Oleate, 2—Polyunsaturated lipid, 3—Polar lipid, 4—Nucleic Acids and Proteins, 5—Proteins, 6—Alkyne cholesterol, 7—Cholesterol. There were also broad background spectral components without any substantially biological spectral features. The full list of spectral components is given in Supplementary Fig. 1. The accuracy of the analysis is clear from the negligible residual (Supplementary Fig. 2). **e** A pictorial depiction of the modified MCR-ALS routine used in this study. A linear combination of the separated profiles (background and signal) gives the total Raman spectrum.

A white light image of one of the HuH7 cells containing LDs is provided in Fig. 1a. LDs are discernable in the figure because of their globular appearance (slight distortion in shape may be due to cell fixing and placing of a cover slide). The triple bond stretching vibration of alkyne cholesterol appears in an isolated region (2119 cm^−1^) of the Raman spectrum allowing an accurate estimation of its distribution in the cell. The Raman image thus obtained has high resemblance to the LDs found inside the cells (Fig. 1a–c). This indicates that alkyne cholesterol is highly concentrated in LDs. We found that the perimeter obtained after ~20% intensity thresholding agrees well with the LDs boundaries seen in the white light image (Fig. 1b). Thus, we could estimate the total area occupied by LDs in each cell (total area of LDs in a cell/cell area). Our analyses indicate a higher proportion of LDs in cells cultured under OL-feed condition (~2 times) compared to CH-feed.

### Raman spectral decomposition using MCR-ALS

Presence of an isolated C≡C Raman band allowed direct imaging of alkyne cholesterol distribution. However, the fingerprint region <2000 cm^−1^ of the Raman spectrum is highly overlapped. Hence, quantitatively extracting band intensities of other biological components is not easy. Multivariate curve resolution (MCR) is a useful method to effect spectral unmixing^27–29^. Since the weak Raman signals are often mixed with highly fluctuating strong background signals, arriving at proper MCR solution is not easy. We have addressed this issue by avoiding all post-acquisition spectral modification and solving for backgrounds or Raman signals focusing on one at a time (codes were written in *Python)*. In the new routine, starting with SVD guess for number of components, we keep modifying the number of components (**k**) in MCR (see “Methods”) until broad signals separate out. However, in doing so Raman spectral components often gets scrambled, which we neglect in the initial cycles. Then, broad spectral components identifiable as background were fed into a new MCR cycle and solved for Raman signals. In this cycle, (a) **k** value is changed until a reasonable solution is obtained with constrained background signal components and then (b) the constraints on the background signals are moderately relaxed and solutions are further optimized. These steps are repeated until a stable solution is obtained such that thereafter no appreciable modifications of the spectral profiles occur. At this stage Raman images are inspected to confirm acceptability. Known background signals (e.g., from glass) are given as initial inputs to speed up the process of background separation. This modified MCR-ALS routine was applied to a large dataset of 91,220 Raman spectra recorded from six different HuH7 cells cultured under two different feed conditions. This big-data was used as a single matrix (A; see “Methods”) to perform MCR-ALS based dimensionality reduction to retrieve relevant spectral components and the corresponding concentration matrix. The full list of spectral components obtained from MCR-ALS is provided in Supplementary Fig. 1. The accuracy of the presented model is evident from the negligible MCR-ALS residual (Supplementary Fig. 2). Seven statistically significant Raman spectral components obtained from MCR-ALS is provided in Fig. 1d and a pictorial depiction of the modified MCR-ALS routine is given in Fig. 1e. Each one of these spectral components was assigned to a specific molecular species based on the characteristic signatures in them (see “Methods” for details). H matrix from MCR-ALS gives Raman images of corresponding spectral components.

## Discussion

MCR components 1, 2, and 3 (Fig. 1d) are lipid spectra and we have assigned them as follows. Component-1 as oleate, component-2 as polyunsaturated lipid and component-3 as polar lipid. In these lipid spectra, the intensity of the Raman band at 1658 cm^−1^ indicates extent of unsaturation (C=C) in the lipid^33^. Polyunsaturated lipid component exhibits higher intensity at 1658 cm^−1^. Polar lipid component has relatively lesser degree of unsaturation and a band at around 720 cm^−1^ (choline group) indicating it polar nature (e.g., phosphatidylcholine)^33^.

**Fig. 2:**
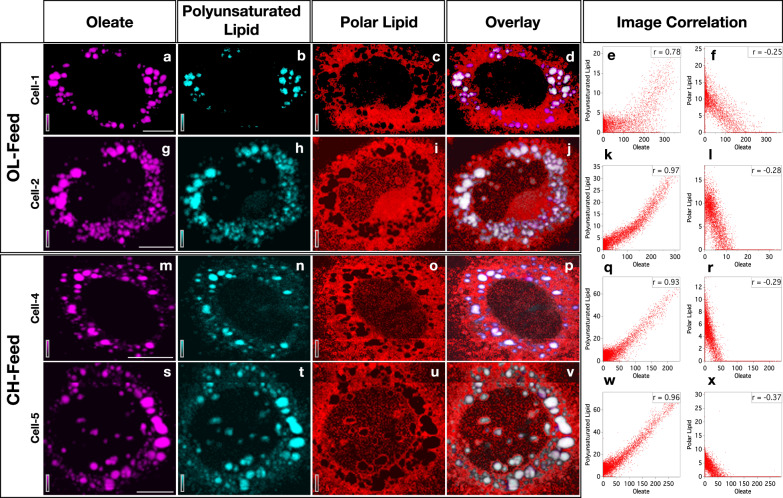
Distribution of lipids in HuH7 cells. Each row represents Raman images from one cell examined. The cell-1 and cell-2 were cultured under OL-feed and the cell-3 and cell-4 under CH-feed. Each column represents distribution of one specific molecular component as indicated at the column header: images in the first column (**a**, **g**, **m**, and **s**) represent the distribution of oleate in the corresponding cells. Overlay of lipid distributions are provided in the fourth column (**d**, **j**, **p**, and **v**). Oleate (**a**, **g**, **m**, and **s**) and polyunsaturated lipid (**b**, **h**, **n**, and **t**) are richly localized in LDs while polar lipid is distributed in the cytosol (**c**, **i**, **o**, and **u**). Images from four representative cells are shown here (see Supplementary Fig. 3 for full list). Pearson image correlation plots for lipids are shown in the last two columns (**e**, **k**, **q**, **w**, **f**, **l**, **r**, and **x**). Correlation coefficients are indicated as insets in each plot. Scale bar 10 μm.

MCR-ALS results also gives spatial distribution of individual spectral components (H matrix; see “Methods”). Spatial distribution (Raman images) of the three lipid-species are provided in Fig. 2 (see also Supplementary Fig. 3). Oleate (Fig. 2a, g, m, s) and polyunsaturated lipid (Fig. 2b, h, n, t) are richly localized in LDs. Polar lipids (Fig. 2c, i, o, u) are found distributed in cytosol medium. In order to better understand the spatial correlation between individual lipid distributions, pixel-to-pixel intensity correlation was performed (images display contrast). Pearson correlation plots^34^ for lipids are shown in Fig. 2e, k, q, w, f, l, r and x (see also Supplementary Figs. 4 and 5). Data points occupying diagonals indicate a perfect correlation in Pearson’s intensity-intensity correlation plots^34^. The corresponding Pearson’s correlation (PC) coefficients are given as inset. Perfectly oppositely correlated images give a PC value of −1 while PC = 1 indicates a perfect positive correlation^34^. It can be seen that distributions of oleate and polyunsaturated lipid are spatially well correlated (Fig. 2e, k, q, w). Negative correlation between oleate and polar lipids (Fig. 2f, l, r, x), on the other hand, reconfirms the spectral assignment. The difference in polarity leads to their mutually exclusive spatial distribution. Further, a good correlation between distributions of alkyne cholesterol and oleate is also found (Supplementary Table 1). Our data indicates that cores of LDs are rich in non-polar lipids and sterol esters.

**Fig. 3:**
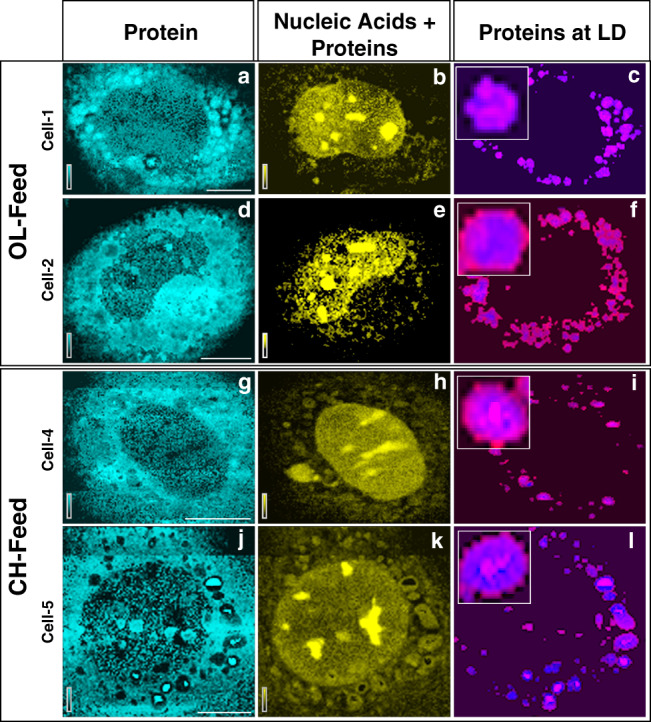
Distribution of proteins and nucleic acids in HuH7 cells. Distribution of proteins (**a**, **d**, **g**, and **j**) and a mix of nuclear proteins + nucleic acids (**b**, **e**, **h**, and **k**) in HuH7 cells. Each row represents Raman images from one cell examined and each column represents the distribution of one molecular component indicated at the column header. Proteins colocalized with LD are shown in the fourth column (**c**, **f**, **i**, and **l**). The location of LDs (blue) was determined as described in the text. Images from 4 representative cells are shown here (see Supplementary Fig. 6). Scale bar 10 μm.

MCR components 4 and 5 are nucleic acids and proteins respectively^35^. MCR component 4, has a spectral marker band for nucleic acids at 785 cm^−1^ (cytosine ring breathing mode)^36^ in addition to prominent protein bands. It means there are some protein species that coexist with nucleic acids. The spatial distribution of these two components in different cells are shown in Fig. 3 (see also supplementary Fig. 6). Proteins are distributed in cytosol (Fig. 3a, d, g, j) while nucleic acids (Fig. 3b, e, h, k) are rich in the nuclear region. Ring like and patchy appearance of proteins are also noticed on LDs surfaces (Fig. 3c, f, I) indicating proteins associated with LDs^1,12^. The technique, however, does not have adequate spatial resolution to investigate proteins exclusively attached to the LDs monolayer surface^37^. Hence, we think that the estimate of Raman intensities of proteins at LDs gives an overestimate of its concentration (Spatial resolution is ~300 times larger than the LDs surface membrane dimension). But the relative variation in the observed protein intensity may reflect the trend of LD-to-LD composition variation. However, a possibility of aggregation (not membrane bound) of protein around LDs may not be completely ruled out (Fig. 3a). Further, dense aggregates of proteins and nucleic acids are observed within the nuclear region (Fig. 3b, e, h, k; Bright yellow). Based on the size of these aggregates and earlier reports^38^, we assign these as nucleoli. More studies are required to completely understand the nature of these aggregates.

Raman spectra of alkyne cholesterol and cholesterol got separated (Fig. 1d; components 6 and 7) in the MCR analysis. This permits a comparison of their relative spatial distribution simultaneously. A band at 1730 cm^−1^ in these spectral components indicates that the sterols are present as esters in the cells^33^. That is, both the sterol derivatives from the feed get enzymatically converted into the corresponding esters inside the cells (Supplementary Fig. 7). Hereafter the names cholesterol and alkyne cholesterol indicate corresponding ester forms unless otherwise specified. When referring to the non-esterified sterols “free form” will be provided in parenthesis after corresponding sterol names.

As discussed earlier, alkyne tagging is one of the simplest structural modifications to a biological molecule such that it behaves identical to the natural analogue. This tag can then be used to attach a fluorophore to visualize molecules using fluorescence spectroscopy^15–17^. In the present study, we intended a critical evaluation of biological behavior of cholesterol and alkyne cholesterol.

Several possible scenarios can be expected to occur during the cell culture (Table 1): (a) cells uptake the sterols (free form) irrespective of the structural modification, but alkyne cholesterol is kept isolated in LDs, (b) when both cholesterol (free form) and alkyne cholesterol (free form) are simultaneously provided in the feed, a preferential uptake of cholesterol (free form) by the cells occur, or (c) the cells treats both cholesterol and alkyne cholesterol equally. In order to successfully evaluate which of these actually occurs, it is important to selectively detect these structural analogues in the same cell under the same conditions. Under OL-feed condition, only alkyne cholesterol (free form) is provided in the culture medium and under this feed condition any cholesterol detected inside the cell is the cholesterol naturally existing in the cells. Analysis of Raman images of the cells cultured under this condition will then permit evaluating whether alkyne cholesterol is completely sequestered in LDs or not. Under the CH-feed condition, on the other hand, both the sterols (free form) are provided in the culture medium such that their uptake by the cell and spatial distribution inside the cell can be compared. Since MCR-ALS analysis has separated the spectra of these sterol derivatives, we could perform such a comparative study accurately.

**Fig. 4:**
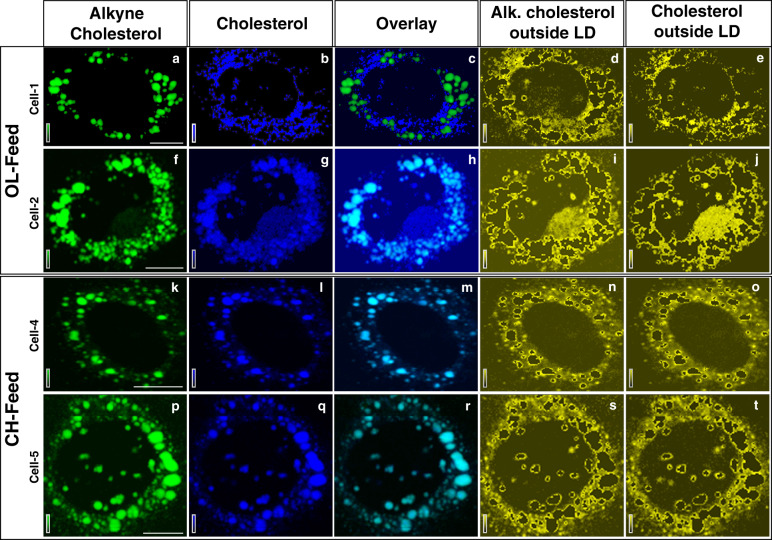
Distribution of sterols in HuH7 cells. **a** Distribution of alkyne cholesterol (**a**, **f**, **k**, and **p**) and cholesterol (**b**, **g**, **l**, and **q**) in HuH7 cells. Each row represents Raman images from one cell examined and each column represents the distribution of one molecular component indicated at the column header. The Raman images of sterols outside LDs (yellow) are shown in the last two columns: alkyne cholesterol (**d**, **i**, **n**, and **s**) and cholesterol (**e**, **j**, **o**, and **t**). Identical distribution can be noted. Images from four representative cells are shown here (see also Supplementary Fig. 8). Scale bar 10 μm.

The spatial distributions of alkyne cholesterol (green) and cholesterol (blue) are given in Fig. 4 (see also Supplementary Fig. 8). At first glimpse it appears that the sterol derivatives have different spatial distributions. Under OL-feed condition, alkyne cholesterol is found inside LDs (Fig. 4a, f) while cholesterol is also found outside (Fig. 4b, g) LDs. On the other hand, in the Raman images of the cells cultured under CH-feed, both the sterols are seen co-localized in LDs (Fig. 4k, p, l, q). It should be noted, however, that these images represent contrast of Raman intensities. That is, high intensity of sterol esters inside LDs makes their distribution outside LDs less apparent in the images. That is depending on the concentration of sterols in LDs, the images may appear different and may not completely depict the true scenario. In order to gain a better understanding of their relative spatial correlation, sterol distributions inside and outside LDs must be separately analyzed. Such an analysis was performed and the distributions of sterols outside LDs are given in Fig. 4d, i, n, s, e, j, o, and t. Remarkable similarity between the distributions can be seen from the plots. Distribution of sterol derivatives outside LDs shows high correlation with an average Pearson Correlation coefficient of 0.79 (Supplementary Table 1). Thus, we show that the spatial distributions of these sterols inside HuH7 cells are identical within the experimental limitation of spatial resolution. Further, our results indicate that the distribution of cholesterol inside LDs depends on the feed condition. Absence of cholesterol (free form) in the OL-feed leads to its poorer concentration inside LDs. However, both sterols are abundantly present inside LDs (Pearson Correlation coefficient >0.9; Supplementary Table 1) in cells cultured under CH-feed because both are provided in the feed.

**Fig. 5:**
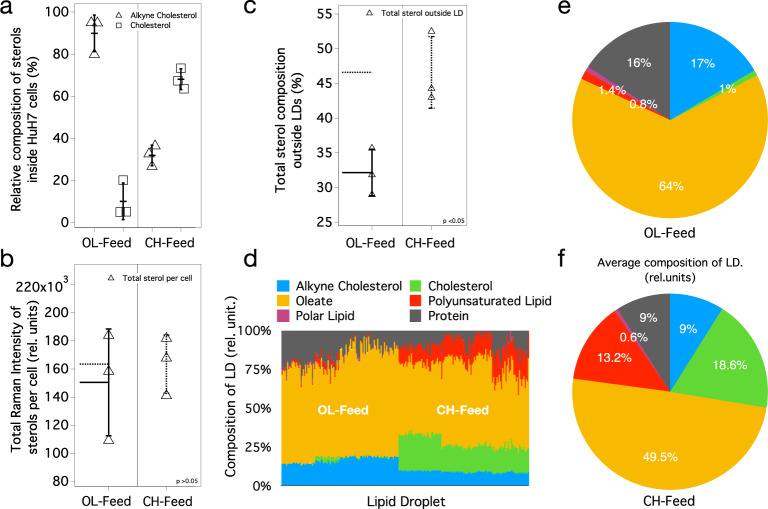
Results of molecular Profiling of LDs. **a** Percent compositions (Raman intensities) of alkyne cholesterol and cholesterol inside HuH7 cells (relative to total sterol) under two different feed conditions. The error bars (vertical lines) represent standard deviations and the mean value is denoted by a horizontal line. Sample size, *n* = 3. **b** Total Raman intensities of sterols (cholesterol + alkyne cholesterol) inside the whole cell (*p* = 0.64); total sterol uptake is comparable under two feed conditions. Sample size, *n* = 3. **c** Total sterol composition outside LDs under two feed conditions. Statistically significant (*p* = 0.02) excess composition of sterols was found outside LDs under CH-feed. Sample size, *n* = 3. **d** Molecular composition of individual LDs under two culture conditions. The height of each colored bar represents area normalized Raman intensity inside each LD (in % scale). The pie charts **e** and **f** show the average values of area normalized total Raman intensities of individual molecular components under two different feed conditions. The Raman intensities are proportional to concentration and can be converted to absolute concentrations by knowing Raman cross-sections. See “Methods” for details of estimating the presented values.

Identical spatial distribution and esterification inside the cells point to the most-probable biological equivalence of these sterol derivatives. However, this should be further verified by comparing their total uptake by the cells. Total Raman intensities of MCR spectral components (${\sum} {H^c}$; see “Methods”) reflects the concentration of these components in cells. The average values of percent composition of sterol components (e.g., %Alkyne cholesterol) inside the cells are shown in Fig. 5a (see also Source Data 1). Under OL-feed condition, ~90% of the total sterol in LDs is alkyne cholesterol. This is because, under this culture condition, only alkyne cholesterol (free form) is provided in the culture medium (Table 1). Cholesterol observed under this condition is the naturally existing cholesterol in the cells. On the other hand, under CH-feed condition 2:1 cholesterol to alkyne cholesterol ratio is observed (Fig. 5a). It agrees with the composition of cholesterol (free form) and alkyne cholesterol (free form) in the culture medium (2:1; see Table 1). The total Raman intensities of sterols per cell (Fig. 5b) are nearly the same (*p* = 0.64). However, the total composition of sterols outside LDs (Fig. 5c) shows significant (*p* = 0.02) difference depending on the feed condition. We have shown that, (1) both sterol derivatives (free form) taken up from the medium gets enzymatically esterified inside HuH7 cells, (2) the spatial distribution of these sterols are identical and (3) the total uptake of sterols by the cells is comparable and is in accordance with the feed composition. Thus, we infer that attaching a triple bond at the end of alkyl-chain of cholesterol does not considerably alter its cellular behavior. This also confirms that the Raman intensities observed in our experiments can account for the molecular composition of the cell (also LDs).

Quantifiability of the MCR concentration profiles evident from the analysis discussed above gives confidence to analyze molecular concentration profiles inside each LDs present in HuH7 cells. To do this we have estimated the total intensity of each molecular component inside each LDs (229 individual LDs) from the corresponding concentration matrices (H). Raman intensities within the LDs locations are only used in this analysis. Area normalized compositions of LDs are shown in Fig. 5d (see also Source Data 1), where each vertical bar plot represents composition of each LDs. Average value of these compositions under OL and CH feed composition are given in Fig. 5e, f respectively. Under OL-feed condition LDs have on an average 1% cholesterol and 17% alkyne cholesterol (6:94). Similarly, under CH-feed condition average sterol composition of LDs changes to ~19% cholesterol and 9% alkyne cholesterol in agreement with the 2:1 feed composition. About 64% of the total composition of LDs is lipid (under both feed conditions). Protein composition is found to be different (16% and 9%) under two feed conditions. Since we believe these numbers are over estimated due to limited spatial resolution, it is not possible to interpret the observed differences. However, a considerable protein composition variation can be expected on LDs. Oleate composition reduces from 64% under OL-feed to ~50% under CH-feed (Table 1). Importantly, the polyunsaturated lipid composition is substantially affected by the feed condition. Under OL-feed condition, only ~1% of polyunsaturated lipid is observed inside LDs (Fig. 5e). Under CH-feed condition, however, an order of magnitude increase in polyunsaturated lipid composition is observed (Fig. 5f) inside each cell. We find this behavior is strongly correlated to the total sterols found outside LDs (Fig. 5c; total sterol composition per cell is statistically similar): the total composition of sterols outside LDs (Fig. 5c; Source Data 1) increases significantly (*p* = 0.02) under CH-feed (~46%) compared to OL-feed (~32%). This higher concentration, we believe, in turn triggers an inflammatory response (production of polyunsaturated lipids^39,40^) in the cell^39–41^. Nearly half of the polyunsaturated lipid produced in the cell is found stored in LDs (Supplementary Fig. 9). Thus, it is clear that the composition of LDs changes depending on the physiological condition of the cell.

We have demonstrated the effective use of Raman spectroscopy and MCR-ALS for the simultaneous imaging of multiple molecular species inside HuH7 cells. We have analyzed a big-data collection of 91,220 spectra from 6 different HuH7 cells. Our MCR model successfully explains all the spectral variations in the dataset (negligible residuals). Raman imaging could locate and quantify the proportion of LDs in HuH7 cells under different feed conditions; such a quantification has relevance in the disease conditions^42,43^. Previous Raman imaging studies have also shown the capability of the technique to detect LDs in cells by mapping lipids, composition of unsaturated lipids under different conditions, etc^44–48^. However, molecular mapping of many different constituents of LDs has never been demonstrated. In our study, seven different molecular species were identified. Three structurally different lipid species have been spectrally isolated, and the relevance of the corresponding relative compositions has been discussed. By simultaneously providing cholesterol (free form) and alkyne cholesterol (free form) in the culture medium, we could compare the biological behavior of the native and structurally modified sterol derivatives. We confirmed enzymatic esterification of sterols inside cells, identical spatial distribution, and nondiscriminatory cellular uptake. Our data shows a high correlation between the concentration of sterols located outside-LDs in HuH7 cells and the over-expression of polyunsaturated lipids. We could clearly identify the changes caused by differences in the composition of culture medium and those caused by biological responses. Raman imaging with MCR-ALS has enormous potential to be used for molecular profiling.

## Methods

### Cell culture and biological sample preparation

*Collagen coating of the glass bottom chamber for cell culture*: cell matrix type IV (Nitta Gelatin Inc., Osaka, Japan) containing collagen was diluted with 10% hydrochloric acid. A total of 1 mL of this diluted collagen solution was added into the glass bottom chamber so that the solution uniformly covers the surface. After a small waiting period, the solution was carefully removed. The chamber was then incubated for about 30–60 min at room temperature. Then the chamber was washed with the medium.

*Cell culture:* HuH-7 (JCRB0403) were commercially purchased. Cells were not tested for mycoplasma contamination and no additional authentication was performed. HuH-7 (JCRB0403) cells were first cultivated in a culture flask using DMEM (Gibco, 11965092) medium at 37 °C. The culture was supplemented with 10% (v/v) lipid-free fetal bovine serum and 5% (v/v) CO_2_. The cells were then (1–2 × 10^5^ cells/mL) inoculated in a glass bottom chamber (Thermo) coated with collagen. After a day selected cells were subjected to two different feed conditions of OL-feed and CH-feed.

**Fig. 6:**
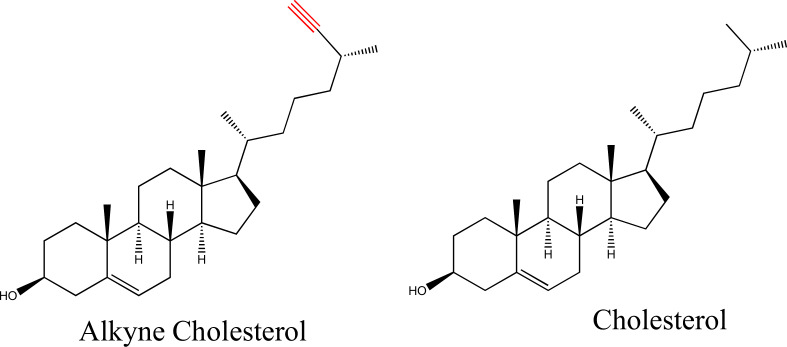
Chemical structures of the sterols. Molecular structures of alkyne cholesterol and cholesterol.

*Feed conditions:* in one feeding condition, named as OL-feed, 50 μM oleic acid was fed on the second day. A mixture of 50 μM oleic acid and 50 μM alkyne cholesterol (Fig. 6) was fed on the third day. In another feed condition, named CH-feed, 50 μM cholesterol (Fig. 6) was fed on the second day instead of oleic acid. A mixture of 50 μM cholesterol and 50 μM alkyne cholesterol was fed on the third day.

*Cell fixing:* On the fourth day, the cells were washed with PBS and 1 mL 4% paraformaldehyde (PFA) was added. It was then incubated for 30 min. PFA was then removed by washing with PBS followed by deionized water.

### Raman spectroscopy

Raman microspectroscopic imaging measurements were carried out with a laboratory-built confocal Raman microspectrometer. A 632.8 nm line of a He-Ne laser (HRP350-EC—HeNe Laser, THORLAB, USA) was used as the laser source. The laser beam was focused into the sample with a 100×(1.4 NA) objective lens (Plan Apo VC; Nikon Corporation, Tokyo, Japan) mounted on an inverted microscope (ECLIPSE Ti; Nikon Corporation, Tokyo, Japan). The back-scattered Raman light was collected with the same objective lens and measured with a spectrometer (MS3504i, 600 lines/mm; SOL Instruments, Ltd., Minsk, Republic of Belarus) and a CCD detector (Newton DU920-M; Andor Technology Plc., Antrim, UK). The laser power at the sample was 15 mW. A piezoelectric stage (custom-made; Physik Instrumente GmbH and Co. KG, Karlsruhe, Germany) was used to carry out Raman mapping measurements (0.3 μm step size). The exposure times were 0.2–0.3 s per point of the cell. Raman images were normalized for laser intensity fluctuation between imaging experiments using indene as external calibration. Spectral, Raman image processing (interpolation, thresholding, area calculation etc.), Pearson’s correlation analyses and detailed image analysis were performed with codes written in *Igor Pro*. Through image analysis we separated LD regions from the Raman images of HuH7 cells (see Fig. 1b in the main manuscript). Then a ratio of the total area of LDs to the total area of cells was estimated. Images were plotted with *Image J*.

### MCR-ALS and SVD

Multivariate curve resolution (MCR) by alternating least squares (ALS) is effective in identifying unique spectral components whose linear combinations constitutes the original data matrix. An original data matrix (A) can be decomposed into spectral components (W) and their concentration profiles (H) as given below^27^.

1 $${\mathrm{A}}_{{\mathrm{m}},{\mathrm{n}}} = {\mathrm{W}}_{{\mathrm{m}},{\mathrm{k}}}{\mathrm{H}}_{{\mathrm{k}},{\mathrm{n}}} + {\mathrm{E}}_{{\mathrm{m}},{\mathrm{n}}}$$

E is the error (or residual). In the present case, A (m × n matrix) consists of n spectra from different spatial locations each with m data points. In this method, the matrix A is decomposed to a matrix W with m × k dimensions, whose columns represent pure component spectra, and another matrix H with k × n dimensions, whose rows represent the intensity profiles of individual spectral components. MCR-ALS is performed iteratively by minimizing the error E such that the Frobenius norm | |A – WH | | ^2^ is minimized. Non-negativity constraints W ≥ 0 and H ≥ 0 are applied during the minimization procedure to obtain physically meaningful solutions. These constraints arise from the fact that neither the Raman spectra nor the concentration profiles will have negative values. In order to solve the MCR equation appropriately, the value of ‘k’ needs to be known or guessed. The number of independent spectral components can be obtained (at least as an initial guess value) by employing singular value decomposition (SVD). In SVD, original m × n matrix is decomposed into UΣV^T^ (U is m × m, Σ is m × n, and V^T^ is n × n), where Σ represents the singular values (diagonal matrix). The number of dominant singular values gives the number of spectral components to be used for the MCR analysis. Remaining components necessarily will be noise. Therefore, SVD can also be used for noise reduction in the data with poor S/N ratio. This can be done by reconstructing the original data (e.g., A) as UΣV^T^ where singular values for all the noise components are kept zero.

The initial values of W and H matrices are also unknown to begin the calculations. We need to provide suitable initial guess values to optimize the decomposition. The SVD spectral components (U) or random numbers can be used as the initial guess spectra (initial W values). Then, the iterations to solve for W and H are performed alternately to arrive at the acceptable final solutions where residual is close to zero. It is possible that such a solution is not completely acceptable due to mixing of spectral components or incomplete removal of background etc. Under such situations, sparser solutions can be sought introducing regularization schemes such as L1 norm (Lasso regression; α) or L2 norm (Ridge regression; β). L1 norm can be applied to H matrix or W matrix depending on situation as given below. If the concentration profiles show mixing of components, L1(H) can be applied where as L1(W) regularization can be applied when mixing of spectral components are observed^27^.

2 $$({\mathrm{W}}^{\mathrm{T}}{\mathrm{W}} + {\upalpha}^2{\mathrm{F}}){\mathrm{H}} = {\mathrm{W}}^{\mathrm{T}}{\mathrm{A}}$$

3 $$({\mathrm{HH}}^{\mathrm{T}} + {\upalpha}^2{\mathrm{F}}){\mathrm{W}} = {\mathrm{HA}}^{\mathrm{T}}$$

Where F is a k × k matrix in which all its elements are unity.

L2 norm can similarly be applied to H matrix or W matrix depending on situation as follows.

4 $$({\mathrm{W}}^{\mathrm{T}}{\mathrm{W}} + {\upbeta}^2{\mathrm{I}}){\mathrm{H}} = {\mathrm{W}}^{\mathrm{T}}{\mathrm{A}}$$

5 $$({\mathrm{HH}}^{\mathrm{T}} + {\upbeta}^2{\mathrm{I}}){\mathrm{W}} = {\mathrm{HA}}^{\mathrm{T}}$$

where I is identity matrix.

The optimized H matrix can be plotted as a 2D image representing the spatial distribution and the spectrum can be used to identify the chemical component.

### Estimating compositions from MCR results

The H matrix in Eq. 1 gives composition information of chemical components (W) for all the cells used in the present study. Values presented in Fig. 5a–d were estimated using the following equations. Percent composition of cholesterol inside each cell was estimated using Eq. 6; where H^c^ is a matrix that denotes H-matrix values corresponding to a single cell (${\sum} { - summation\,of\,all\,the\,elements\,in\,the\,matrix}$). Similarly, percent composition of alkyne cholesterol inside the cells was estimated using Eq. 7. The sum of H^c^ values for cholesterol and alkyne cholesterol gives total Raman intensity of sterols in a given cell (Eq. 8). The percent composition of sterols outside LDs in each cell was estimated using Eq. 9; where H^LD^ denotes H-matrix values corresponding to a single LD (an image region of a specific LD) inside a cell.

Each vertical bar in Fig. 5d represents composition of one LD in a cell. Each color code is the percent composition of one chemical component estimated using Eq. 9. For instance, the percent composition of oleate was calculated by substituting ${\mathrm{H}}_{{\mathrm{species}}}^{{\mathrm{LD}}}$ with ${\mathrm{H}}_{{\mathrm{Oleate}}}^{{\mathrm{LD}}}$. Since nucleic acids could not be separated as a pure component in the analysis, it was not included in the LD composition analysis.

6 $$\frac{{100{\sum} {H_{Cholesterol}^c} }}{{\left( {{\sum} {{\mathrm{H}}_{{\mathrm{Alkyne}}\,{\mathrm{Cholesterol}}}^{\mathrm{c}}} + {\sum} {{\mathrm{H}}_{{\mathrm{Cholesterol}}}^{\mathrm{c}}} } \right)}}$$

7 $$\frac{{100{\sum} {{\mathrm{H}}_{{\mathrm{Alkyne}}\,{\mathrm{Cholesterol}}}^{\mathrm{c}}} }}{{\left( {{\sum} {{\mathrm{H}}_{{\mathrm{Alkyne}}\,{\mathrm{Cholesterol}}}^{\mathrm{c}}} + {\sum} {{\mathrm{H}}_{{\mathrm{Cholesterol}}}^{\mathrm{c}}} } \right)}}$$

8 $${\sum} {({\mathrm{H}}_{{\mathrm{Alkyne}}\,{\mathrm{Cholesterol}}}^{\mathrm{c}} + {\mathrm{H}}_{{\mathrm{Cholesterol}}}^{\mathrm{c}})}$$

9 $$\frac{{100\left\{ {\left( {{\sum} {\left( {{\mathrm{H}}_{{\mathrm{Alkyne}}\;\,{\mathrm{Cholesterol}}}^{\mathrm{c}} + {\mathrm{H}}_{{\mathrm{Cholesterol}}}^{\mathrm{c}}} \right.} } \right) - \left( {{\sum} {\left( {{\mathrm{H}}_{{\mathrm{Alkyne}}\,\;{\mathrm{Cholesterol}}}^{{\mathrm{LDs}}} + {\mathrm{H}}_{{\mathrm{Cholesterol}}}^{{\mathrm{LDs}}}} \right)} } \right)} \right\}}}{{{\sum} {\left( {{\mathrm{H}}_{{\mathrm{Alkyne}}\;\,{\mathrm{Cholesterol}}}^{\mathrm{c}} + {\mathrm{H}}_{{\mathrm{Cholesterol}}}^{\mathrm{c}}} \right)} }}$$

10 $$\frac{{100{\sum} {\left( {{\mathrm{H}}_{{\mathrm{species}}}^{{\mathrm{LD}}}} \right)} }}{{{\sum} {\left( {{\mathrm{H}}_{{\mathrm{Oleate}}}^{{\mathrm{LD}}} + {\mathrm{H}}_{{\mathrm{Polyunsaturated}}\;{\mathrm{Lipid}}}^{{\mathrm{LD}}} + {\mathrm{H}}_{{\mathrm{Cholesterol}}}^{{\mathrm{LD}}} + {\mathrm{H}}_{{\mathrm{Alkyne}}\;{\mathrm{Cholesterol}}}^{{\mathrm{LD}}} + {\mathrm{H}}_{{\mathrm{Polar}}\;{\mathrm{Lipid}}}^{{\mathrm{LD}}} + {\mathrm{H}}_{{\mathrm{Proteins}}}^{{\mathrm{LD}}}} \right)} }}$$

where, species = oleate, polyunsaturated lipids, cholesterol, alkyne cholesterol, polar lipid, or proteins.

Compositions of other components were similarly calculated. More than 100 LDs were analyzed from cells cultured under each feed conditions. An average value of composition is plotted as a pie chart and is shown in Fig. 5e, f.

### Statistics and reproducibility

Duplicate culture experiments were conducted. Cells labeled cell-1 and cell-2 were from one OL-feed culture and cell-3 was from a duplicate OL-feed culture. Similarly, cell-4 and cell-5 were from one CH-feed culture and cell 6 was from a duplicate CH-feed culture. Triplicate Raman imaging studies were performed (cells 1, 2, and 3 from OL feed and cells 4, 5, and 6 from CH feed). Technical replicates were performed for a few select cells and the reproducibility of results was confirmed. Statistical analyses were performed with sample size *n* = 3. Two-sided *t*-tests were conducted. For Fig. 5b, degree of freedom was 2 for both OL and CH feed data, *p* = 0.64, *t* = −0.5 and the low confidence intervals for the mean was 147,616 (Mean = 150,469 for OL-feed) and the low confidence intervals for the mean was 166,347 (Mean = 163,494 for CH feed). For Fig. 5c, degree of freedom was 2 for both OL and CH feed data, *p* = 0.02, *t* = −4.1 and the confidence intervals for the mean were 27.3–36.9 (OL feed; Mean = 32.2) and 39.2–53.9 (CH feed; Mean = 46.6).

### Spectral assignments

In MCR components 1, 2, and 3 (Fig. 1d; see also Supplementary Fig. 1) a strong band at 1440 cm^−1^ corresponding to –CH_2_- scissoring mode appears along with prominent bands at 1305 cm^−1^ (CH– bending) and 1080 cm^−1^ (C–C stretching rich in gauche conformation along the carbon chain) are observed. These are characteristic vibrations of alkyl chains and hence these components represent lipids. Further, the band at 1745 cm^−1^ indicates ester carbonyl group of the corresponding ester group. The band at 1658 cm^−1^ is due to unsaturation (C=C) in the alkyl chain. The intensity of this band indicates extent of unsaturation in the lipid. Component_2 has a higher intensity at 1658 cm^−1^ compared to component _1 and component _3 indicating largest degree of unsaturation among the three lipids. Therefore, we assign component_2 as polyunsaturated lipid. A close similarity of component _1 spectral profile to oleate was confirmed by comparing with oleate Raman spectrum^33^. Component _3 spectrum has relatively lower degree of unsaturation and a band at around 720 cm^−1^, which indicates presence of choline functional group. Therefore, we assign component _3 to polar lipids (e.g., phosphatidylcholine).

MCR component 5 shows prominent protein spectral features such as, strong amide-I (peptide backbone vibration) at 1665 cm^−1^, C–H deformation mode at 1450 cm^−1^, tryptophan Cα–H deformation at 1340 cm^−1^, phenylalanine at 1003 cm^−1^. Therefore, MCR spectral component 5 represents proteins in the cell. MCR component 4, on the other hand, has an additional spectral marker band for nucleic acids at 785 cm^−1^ (cytosine ring breathing mode in addition to common protein spectral features) and a weak band at 813 cm^−1^ (phosphodiester vibrational mode). Hence this spectral component has been assigned to a mixture of nucleic acids and proteins^35,36^.

Component_6 and component_7 show characteristic vibrations of sterol ring below 1000 cm^−1^, with prominent bands at 427 cm^−1^ and 702 cm^−1^. Further a strong band at 1438 cm^−1^ corresponding to CH_2_- bending mode and 1670 cm^−1^ marker band corresponding to C=C stretch can be observed. Therefore, we assign these two components as sterols. Further, a band at 2119 cm^−1^ (CC triple bond stretch) distinguishes component_6 from 7. Thus component_6 has been alkyne assigned as alkyne cholesterol and component 7 as cholesterol. Further, a small band at 1730 cm^−1^ (ester C=O stretching) in these MCR components indicates that the sterol derivatives are present as esters.

### Reporting summary

Further information on research design is available in the Nature Research Reporting Summary linked to this article.

## Supplementary information

Supplementary Information

Description of additional supplementary files

Supplementary Data 1

Reporting Summary

## Data Availability

The source data for Fig. 5 is provided in Supplmentary Data 1. The data sets generated during and/or analyzed during the current study are available from the corresponding author on reasonable request.

## Code availability

Software implementing MCR-ALS can be found at https://github.com/mshrAndo/PyMCR.
