# Supplementary material for: Molecular profiling of lipid droplets inside HuH7 cells with Raman micro-spectroscopy
Source: Commun Biol. 2020 Jul 10;3:372. doi: 10.1038/s42003-020-1100-4 (PMC7351753; doi:10.1038/s42003-020-1100-4)
Supplement: Supplementary file 1 — Supplementary Information [file 42003_2020_1100_MOESM1_ESM.pdf]

Supplementary Figures

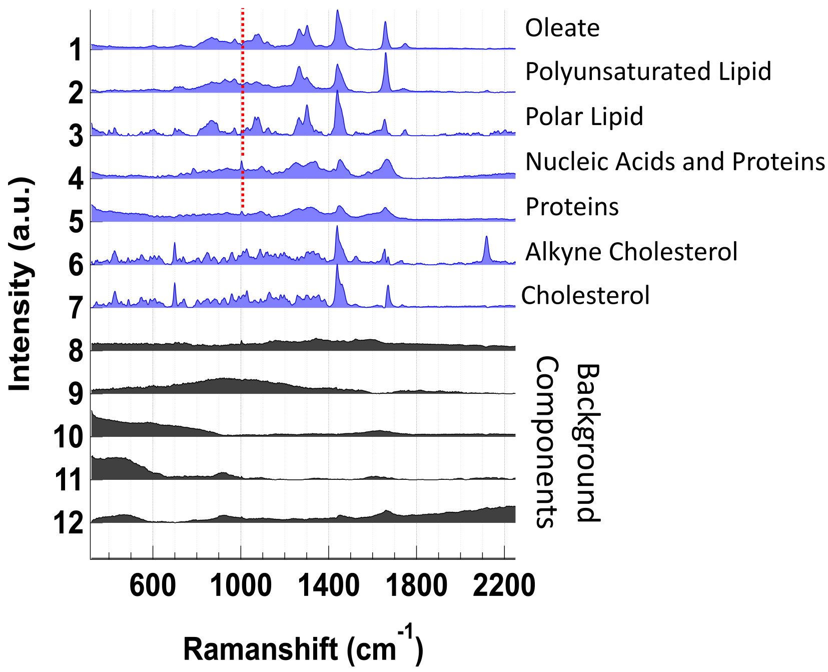

Supplementary Figure 1. Complete list of MCR spectral components and background components (gray). The red line indicates absence of protein signature band at 1003 cm^{-1} in the lipid spectra suggesting a complete separation of these spectral components (*See* "spectral assignments" in Methods).

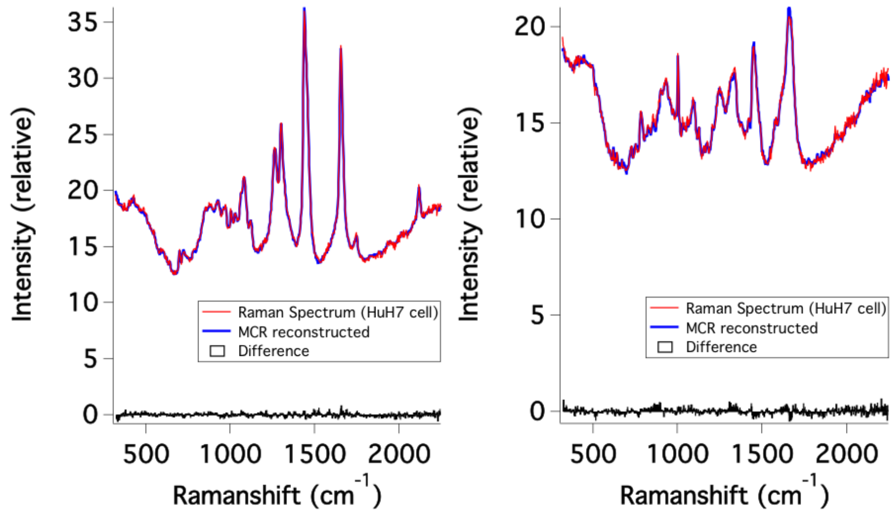

Supplementary Figure 2. Comparison of the signal and the residual in the MCR-ALS analysis.

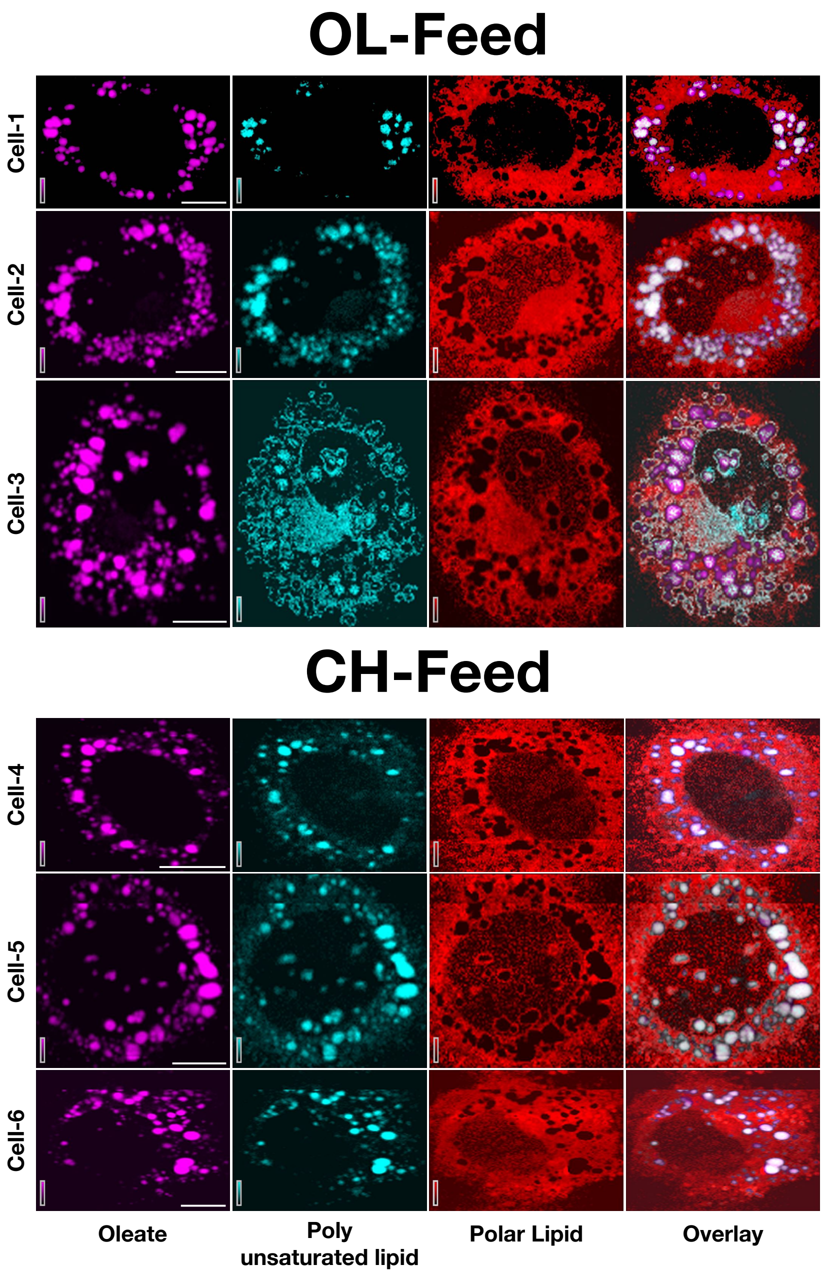

Supplementary Figure 3. Distribution of lipids in the HuH7 cells. Scale bar 10 μm.

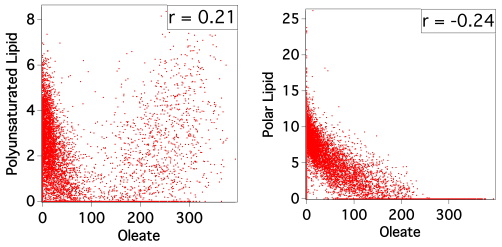

Supplementary Figure 4. Pearsons's correlation between lipid images for cell-3 (Cultured under OL-feed condition).

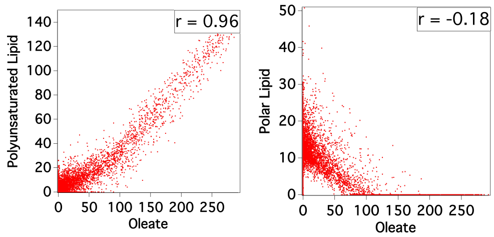

Supplementary Figure 5. Pearson's correlation between lipid images for cell-6 (Cultured under CH-feed condition).

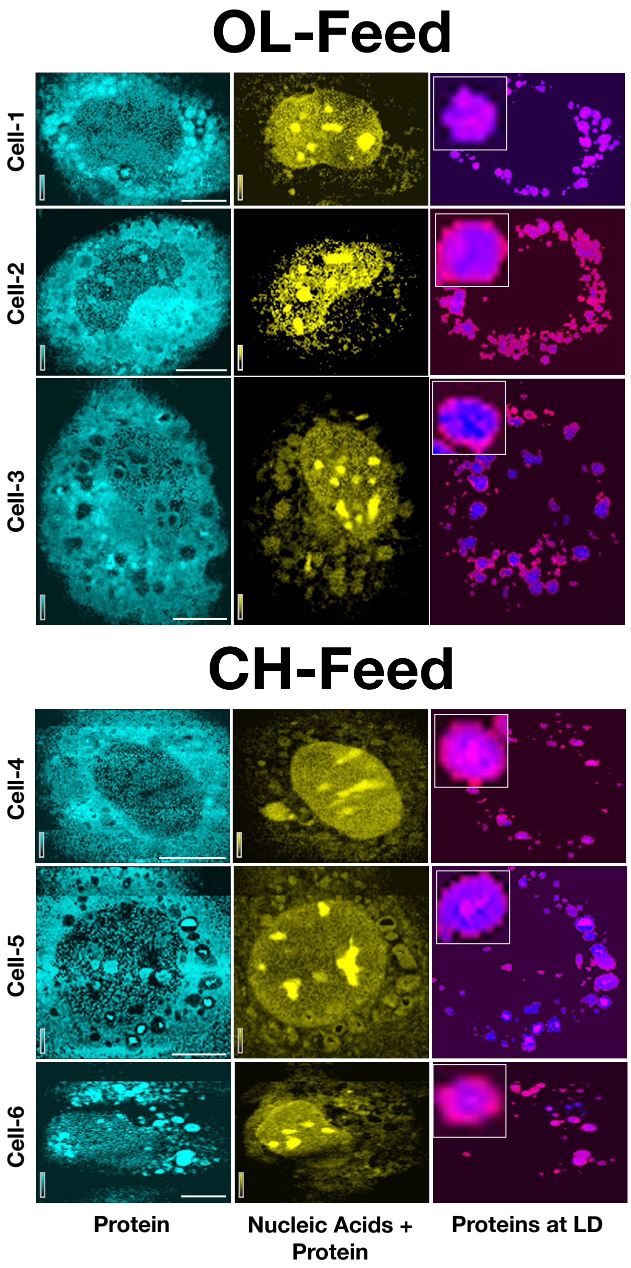

Supplementary Figure 6. Distribution of proteins and nucleic acids in the HuH7 cells. Scale bar 10 μm.

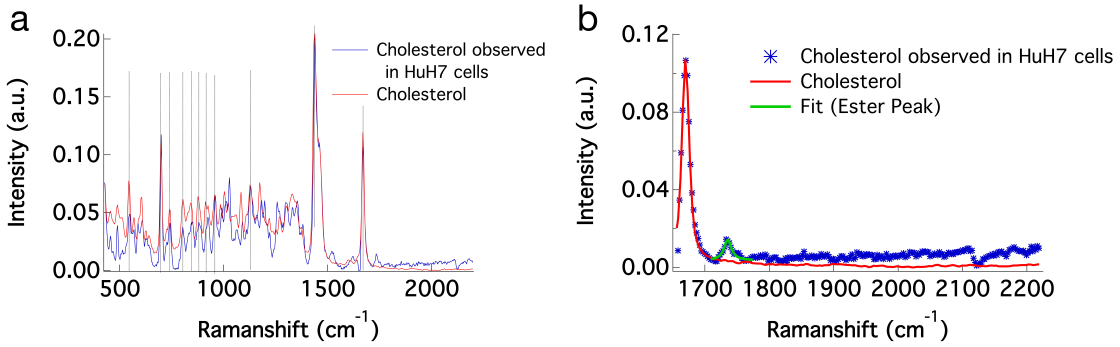

Supplementary Figure 7. a) comparison of Raman spectra of standard cholesterol and cholesterol observed in the cells. b) zoomed plot.

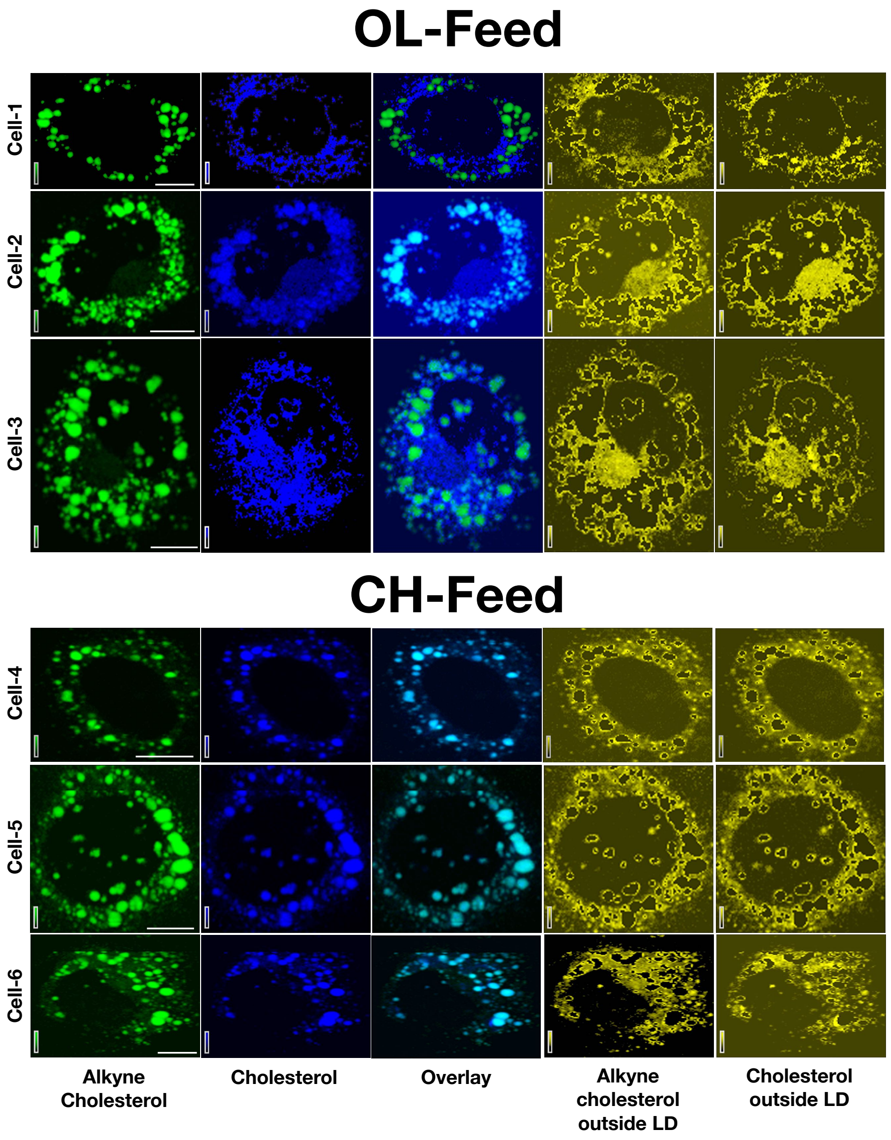

Supplementary Figure 8. Distribution of sterols in the HuH7 cells. Scale bar 10 µm.

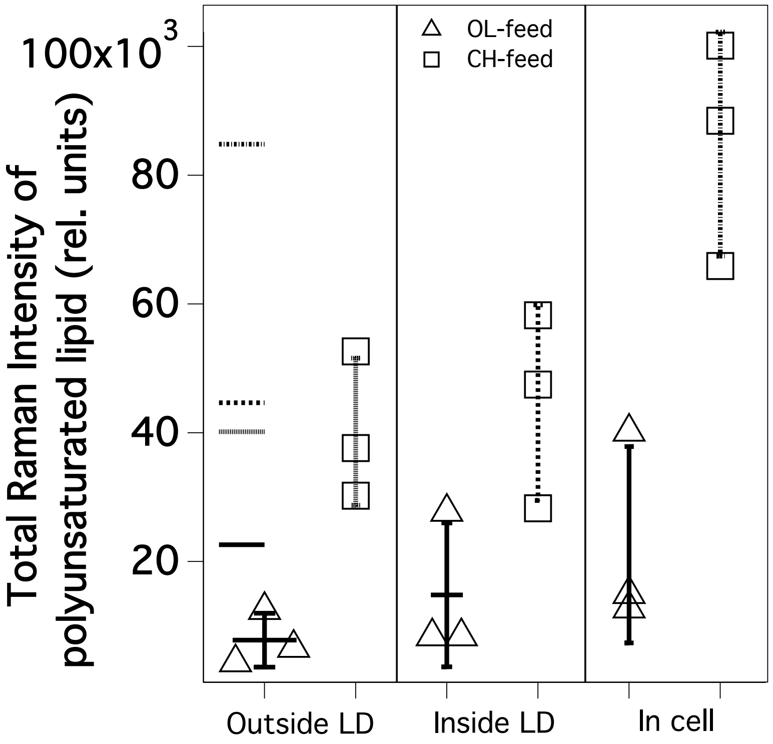

Supplementary Figure 9. Total Raman intensity of polyunsaturated lipid at different locations (e.g. outside LDs) of the cells.

Supplementary Tables

Supplementary Table 1. Pearson's correlates coefficients (PC) for different pairs of Raman images.

Correlation Pair	Pearson's Correlation Coefficient for cells					
	1	2	3	4	5	6
**Lipid 1 & 2**	0.78	0.97	0.21	0.93	0.96	0.96
**Lipid 2 & 3**	-0.12	-0.28	0.42	-0.17	-0.18	-0.13
**Lipid 1 & 3**	-0.25	-0.35	-0.24	-0.29	-0.37	-0.18
**Alkyne Cholesterol & lipid 1**	0.99	0.99	0.99	0.99	0.99	0.98
**Cholesterol & alkyne cholesterol (Outside LD)**	0.63	0.79	0.61	0.96	0.92	0.83
**Cholesterol & alkyne cholesterol (Inside LD)**	0.11	0.95	0.49	0.99	0.99	0.99
