# Supplementary material for: Molecular profiling of lipid droplets inside HuH7 cells with Raman micro-spectroscopy
Source: Commun Biol. 2020 Jul 10;3:372. doi: 10.1038/s42003-020-1100-4 (PMC7351753; doi:10.1038/s42003-020-1100-4)
Supplement: Supplementary file 2 — Description of additional supplementary files [file 42003_2020_1100_MOESM2_ESM.docx]

Description of Additional Supplementary Files

Supplementary Data 1: Source data underlying plots shown in Fig 5.
